# Vanishing Knowledge of Plant Species in the Wadi Allaqi Desert Area of Egypt

**DOI:** 10.1007/s10745-016-9826-9

**Published:** 2016-05-19

**Authors:** Hanaa A. Kandal, Hoda A. Yacoub, Menno P. Gerkema, Jac. A. A. Swart

**Affiliations:** 1 University of Groningen, Groningen, Groningen Netherlands; 2 Wadi Allaqi Biosphere Reserve, Nature Conservation Sector, EEAA, Aswan, Egypt

## Introduction

The distribution and abundance of plant species are strongly linked to the physical environmental and thus to anthropogenic disturbances. Changes in desert vegetation, in particular, can have drastic impacts on human livelihoods in these areas as ecosystem services may be affected (Dubroeucq and Livenais 2004; Klintenberg *et al.*^2007^; Käyhkö *et al.*^2011^; Rohde and Hoffman 2012).

The Wadi Allaqi Biosphere Reserve is a hyper-arid desert in southern Egypt, inhabited by Bedouin. Traditionally, the Bedouin of Wadi Allaqi are a highly mobile group of pastoralists who follow seasonal migration routes taking advantage of local plant species as food, fuel, medicine, construction materials, and fodder for their livestock (Belal *et al.*^1998^; Badri and Hamed 2000). Changes in environmental conditions of the wadi, however, have led to a change in composition of the native vegetation and its utilization by the Bedouin, which in turn has impacted their livelihood patterns (Briggs *et al.*^1993^; Solway and Mekki 1999; Shaltout *et al.*^2010^). The construction of the Aswan High Dam in from the 1960s and the creation of a permanent water resource in the form of Lake Nasser, in particular, have radically altered the natural environment and associated vegetation in this hyper-arid area (Pulford *et al.*^1992^; Springuel 1994; Belal *et al.*^1998^).

Several studies have demonstrated the strong effects of Lake Nasser on the vegetation of the lower part of the wadi system, which drains into the now-inundated Nile valley in Egyptian Nubia (White 1988; Springuel and Murphy 1990; Springuel and Mekki 1994; Briggs *et al.*^1999^; Badri and Hamed 2000; Briggs *et al.*^2003^; Sheded *et al.*^2006^; Shaltout *et al.*^2009^; Yacoub 2009; Shaltout *et al.*^2010^). Many Wadi Allaqi Bedouin have migrated and settled on the shores of the Lake in order to take advantage of this water resource and the newly established grazing areas. As a result, these traditionally nomadic people have adopted a semi-settled pattern of life (Briggs *et al*. 1993; Ali *et al*. 2000).

A more recent development is a settlement in Wadi Allaqi with houses, schools, and health facilities provided by the Egyptian government, creating new livelihood possibilities (Springuel and Belal 2001). As part of this development, Allaqi Village was built in 2003. In this study, we investigate changes in plant species knowledge of the Bedouin community in Wadi Allaqi Biosphere Reserve to assess the effects of the new settlements on the shores of the Lake in the context of the socioeconomic dynamics of the area.

## The Study Area

**Fig. 1:**
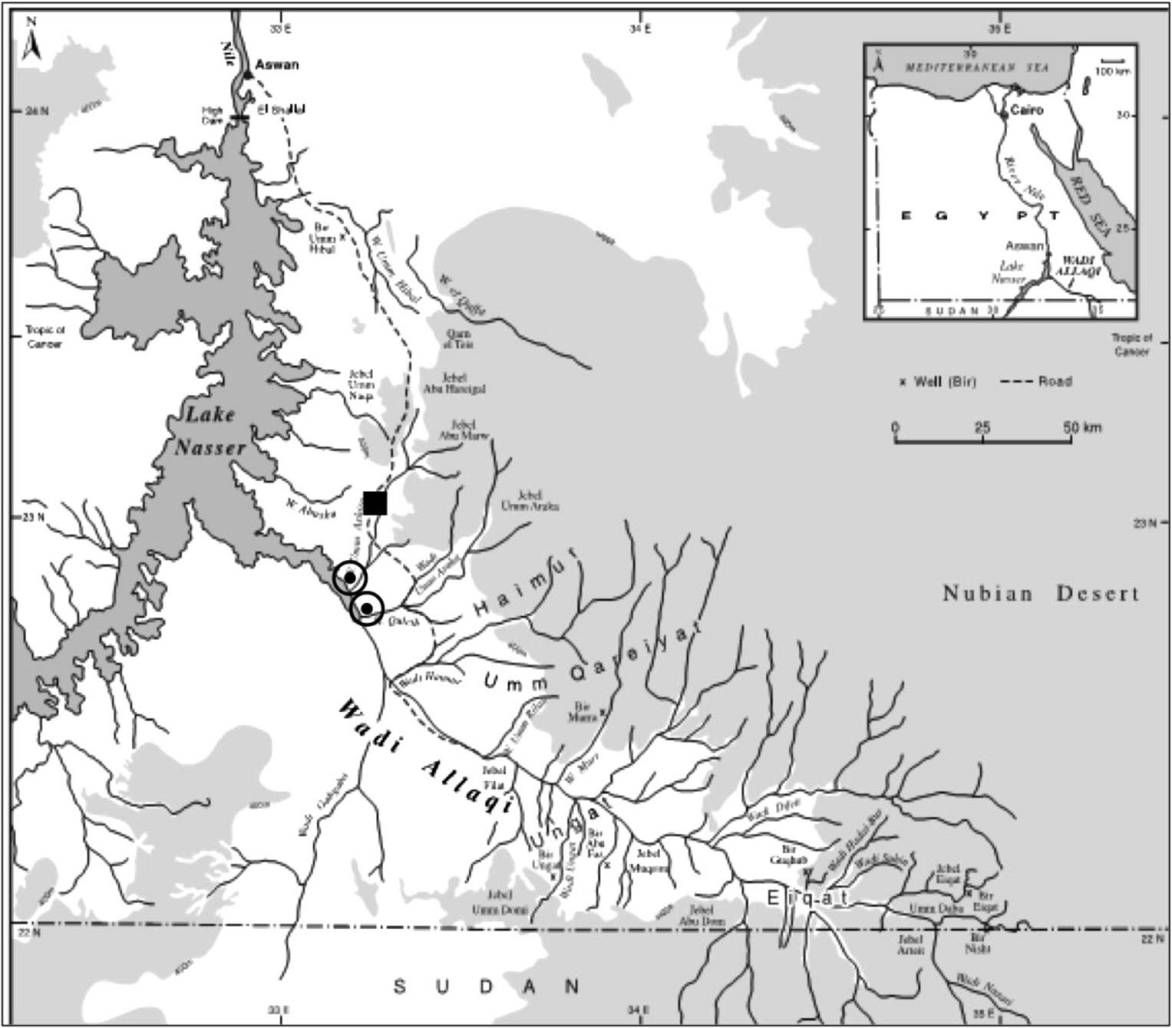
Map of Wadi Allaqi area. Legends: ν: Allaqi Village; /: Shore of Lake Nasser where Bedouin people live

Wadi Allaqi is the largest among numerous wadis dissecting Egypt’s Eastern Desert (Egyptian Nubian Desert) (Kassas and Girgis 1970). The wadi is located at the southern part of the Eastern Desert, situated on the eastern shore of Lake Nasser (Fig. 1), about 180 km south of Aswan (22° and 23°N to 33°and 35°E) (Shaltout *et al.*^2010^). It is a major dry river (desert river) running from the Red Sea hills to the shores of Lake Nasser with a total length of 250 km, approximately 200 km in Egypt and 50 km in Sudan (Badri and Hamed 2000). It has an average width of about 1 km but broadens considerably as it approaches Lake Nasser.

Wadi Allaqi forms one of the most extensive drainage systems in Egypt’s Eastern Desert, collecting runoff water from rare cloudbursts and spells of rain. The area is characterized by a hyper-arid climate with an aridity index of less than 0.05 (Ayyad and Ghabbour 1986; Sheded 1992), and by high variability in the amount and duration of annual precipitation (Ayyad 1973; Ayyad and El-Ghonemy 1976), which averages less than 5 mm annually. Many years may pass without any rain. Its biogeographical characteristics are diverse. One can find tropical biota at the southern, or Sudano-Saharan and Ethiopian zones, and temperate biota at the northern or Mediterranean zone (Belal *et al.*^1998^; Springuel and Belal 2001). The climate has considerable impact on the dynamics of wadi vegetation and species composition and distribution (Kassas 1952; Noy-Meir 1973; Fossati *et al.*^1998^). The natural vegetation of Wadi Allaqi represents a typical desert flora, with a majority of therophyte and chamaephyte species (Sheded 1998; Shaltout *et al.*^2010^).

Two main ethnic groups inhabit Wadi Allaqi: the Ababda and the Bishari or Bisharin, together often referred as ‘Bedouin,’ a term derived from the Arab word *Bedu* meaning inhabitant of the deserts (Yacou 2012). The Ababda have been residents in the southeastern desert of Egypt (Nubian Desert) for at least several centuries (Hobbs 1989; Briggs *et al.*^1993^). The Bishari originally came from the Egypt-Sudan border and Red Sea hills and many of them live now in the upstream Wadi, whereas the Ababda live further downstream (Belal *et al.*^1998^). Our research area is downstream Wadi Allaqi where over the last 50 years both groups have settled on the shores and in the vicinity of Lake Nasser in order to take advantage of this water resource and the new grazing areas established after its formation (1967–1972). In spite of their different ethnic backgrounds, they maintain cordial relations and also intermarry. Reciprocal use of territory is common and no substantial conflicts over resources have yet occurred between them (Belal *et al.*^1998^). The Egyptian government considers them a single community and does not distinguish between them tribes in its settlement policies.

Mohamed *et al.* (1991), from a 1986 census, characterized the area as inhabited by a total of 218 people: 89.5 % Ababda and 10.5 % Bishari. Mekki and Briggs (1991) recorded 220 inhabitants, of which approximately 66 % were Ababda and the remainder Bishari. In 2012, the number of Wadi Allaqi Bedouin in the newly established Allaqi Village was 274 (EEAA 2012). Mohamed *et al.* (1991) noted in their study that population numbers in the wadi have varied as a result of changes in the Lake level and environmental circumstances in the surrounding desert. Further, weather fluctuations during the hot summers and cold winters have an important influence on the relocation of settlements within the wadi.

The main components of the economic system used to be livestock, charcoal production, medicinal plant collection, small-scale cultivation, trade, and wage labor (Briggs *et al.*^1993^; Belal *et al.*^1998^; Briggs *et al.*^1999^). In addition, Wadi Allaqi is home to other groups, such as itinerant fishermen, miners, and farmers, along with military personnel who often have a more permanent residence because of its strategic location along the Egyptian-Sudanese border (Belal *et al.*^2009^).

Environmental and ecological conditions in Wadi Allaqi changed radically after completion of the Aswan High Dam, which was opened in 1970, consequently impacting the available natural resource base, livelihood patterns, and socioeconomic opportunities of the Wadi Allaqi Bedouin (Pulford *et al.*^1992^; Briggs *et al.*^1993^, ^1999^; Sharp *et al.*^2003^).

The ecology of downstream Wadi Allaqi is now dominated by Lake Nasser, which is nowadays a crucial source of reliable water. A narrow inlet of the Lake, named Khor Allaqi, penetrates about 80 km into Wadi Allaqi. Annual fluctuations in lake level create a temporarily inundated area over a width of up to 40 km twice each year. A diversity of ecosystems are found close to the Lake in the transition zone between completely dry land upstream and the permanently inundated land downstream, Khor Allaqi, and in the extreme arid deserts further away from the Lake.

## Materials and Methods

### Data Collection

We carried out interviews with Wadi Allaqi Bedouin at their living locations. In total, nine visits to the Wadi Allaqi area were made in the period 2011 to 2013. The locations visited were Khor Allaqi and the newly established Allaqi Village, located about sixteen km from the Lake. These settlements are comprised of nomadic migrants who arrived after the creation of Lake Nasser (Fig. 1). Those who settled close to the Lake still live in traditional tents and move seasonally through the area. During our field trips we found around 30–40 people in there. People in Allaqi Village live in houses and are more sedentary. Because many Bedouins of Wadi Allaqi continue to move through the desert in response to availability of grazing resources, and employment, or business opportunities, it is difficult to estimate total population size. However, according to the Egyptian Environmental Affairs Agency (EEAA 2012) the Bedouin population in Allaqi Village is around 274.

Information on species knowledge related to their use and livelihood patterns was collected during these visits. Selections of plant species (see Appendix) were made prior to the interviews based on prior observation of use by Wadi Allaqi Bedouin, and referring to Egyptian flora books (Tackholm 1974; Boulos 1999, 2000, 2002) and previously published literature (Goodman and Hobbs 1988; Springuel 1994; Badri *et al.*^1996^; Hamed *et al.*^1996^, ^1997^, ^1999^, ^2004^; Belal *et al.*^1998^, ^2009^; Briggs *et al.*^1999^, ^2007^; Hamed and El-Emary 1999; Badri and Hamed 2000; Ali *et al.*^2001^; Hassan *et al.*^2001^; Briggs *et al.*^2003^; Sharp *et al.*^2003^; EEAA 2005; Sheded *et al.*^2006^; Radwan 2007; Ibrahim and Baker 2009; Yacoub 2009; Shaltout *et al.*^2010^). Samples of plants, plant parts, and images were used for interviewees to identify plant species and provide local names.

The interviews were informal, usually one-to-one, and took place on different occasions. Interviews with females were usually conducted inside houses or tents, while interviews with males took place in designated guest areas at some distance from the private spaces of the household. Some interviews included the participation of neighbors and relatives in the discussions, although individual opinions still were recorded. In total, eight such group sessions on the shores of Lake Nasser and in Allaqi Village were conducted, including 24 respondents with two to five participants per session. Most interviews (individual or in groups) were completed within about an hour, although a few lasted much longer.

During the interviews, questions were generally open-ended. In addition to personal data such as gender and estimated age, and socioeconomic and historical data, most questions related to plant utilization (medicinal use, fuel wood, grazing, and charcoal production), and knowledge and awareness of Allaqi plant species (see Appendix). Because the interviewers were female researchers (Kandal and Yacoub), conversation with Wadi Allaqi Bedouin men was restricted. These interviews were carried out in cooperation with the accompanying male member of the research team.

Most Bedouin in Wadi Allaqi are illiterate and were rather suspicious of foreigners and notation equipment (pen and paper, electronic recording) during the interviews. Therefore, the interviewers were unable to distribute questionnaires and took notes during the interviews or used devices for recording conversations. Detailed information derived from the interviews and focus groups was, therefore, recorded from memory directly after the interview sessions. Consequently, no direct quotations from interviewees could be collected.

### Data Analysis

Based on normalized gender group size (Box 1), the species recognition index (*R*_*s*_) was defined by: *R*_*s*_ *= Σ (N*_*p*_*/N*_*t*_*)/N*_*s*_, where *N*_*p*_*is* number of people that recognizes or know a particular species, *N*_*t*_ is the group size, and *N*_*s*_ is the number of species involved. Based on this procedure, we divided our data into male and female groups, into location groups (living at the Lake and in the Village), and, finally, into age groups (younger than 20 years, 20–29 years, 30–39 years, 40–49 years, 50–59 years, 60–69 years, and 70 years or older). Accordingly, we determined the mean species recognition index for these groups.

Box 1. Normalization procedure of the raw database by using an example

**Table:** 

If 7 out of a group A of 10 people recognize a particular species S, then we may say that the group’s species recognition for S is 70 %. If our group of 10 people consists of 4 men and 6 women, and it appears that all men and 3 women recognize this species, the *R*_*s*_ of the whole group remains 70 %, but that of men is 100 % and that of women is 50 %. For a second group B consisting of 5 men and 4 women, 3 men and 2 women recognize the species S. The species recognition index of group B is thus 62.5 %, and that of men and women is respectively 60 and 50 %. However, we cannot compare A and B directly because they have different gender distributions with a different *R*_*s*_, which may affect the *R*_*s*_ of group B as a whole. Moreover, the sizes of groups A and B also differ. Thus, to compare different groups we have to normalize groups to similar gender distributions sizes. Thus, in our example, we multiply all numbers in our group such that all gender groups have the same size. This results in normalized gender group sizes of 6 people, and a normalized group size of 12 for both *A* and *B*. Using the equation *R*_*s*_ *= Σ (N*_*p*_ */N*_*t*_ *)/N*_*s*_, where *N*_*p*_ *is* normalized number of people in a certain group recognizing a particular species, *N*_*t*_ is the normalized total group size, and *N*_*s*_ is the number of involved species (in this example only one), the species recognition index of species *S* appears to be 75 and 55 % for A and B, respectively, and for men and women (now over both groups) 80 and 50 %. Next we can determine *R*_*s*_ for all species and derive mean *R*_*s*_ for other statistics.	

## Results

We interviewed 94 people (52 women and 42 men), of whom 51 lived in Allaqi Village and 43 on the Lake Nasser shore (Table 1). Thus, we sampled about one-third of the Wadi Allaqi population. Of our list of 95 species, 61 species (64 %) were recognized by at least one respondent (Table 4 in Appendix). Accordingly, we determined the species recognition index of these species for men and women, people living at the Lake and in the Village, and for several age classifications (Table 1).

**Table 1:** Mean values (%) and SEM. (%) of species recognition (*R*_*s*_) across 61 species (*N*_*s*_)

Groups	*N*_*t*_	Mean *R*_*s*_ (%)	SEM (%)
All respondents	94	60.3	4.1
Men	42	65.3	3.95
Women	52	55.2	4.40
Age ≥50 years	31	81.1	3.16
Age <50 years	63	44.6	5.34
Lake Nasser	43	62.7	4.02
Allaqi Village	51	57.9	4.2
Men in Allaqi Village	22	63.1	4.1
Women in Allaqi Village	29	52.7	4.54
Men at Lake Nasser	20	67.5	3.83
Women at Lake Nasser	23	57.8	4.29
Age ≥50 years at Lake Nasser	12	85.4	2.94
Age <50 years at Lake Nasser	31	45.6	5.39
Age ≥50 years in Allaqi Village	19	76.9	3.48
Age <50 years in Allaqi Village	32	43.6	5.29

None of the *R*_*s*_s for the different groups in Table 1 appears to be normally distributed (One Sample Kolmogorov-Smirnov Test in SPSS 22). We therefore applied the non-parametric Samples Wilcoxon Signed Rank test (SPSS 22) to all the paired variables to indicate differences between the species knowledge of the different groups based on gender, age, and location (Table 2). It appears that all data pairs demonstrate a significant difference *(p < 0.000*).

**Table 2:** Mean values (%) and SEM (%) of species recognition (R_s_) for differences from Table 1 across 61 species (*N*_*s*_)

Difference between:	Mean *R*_*s*_ difference (%)	SEM (%)
Men and women	10.0	1.16
Age ≥50 years and age <50 years	36.5	3.70
People at Lake Nasser and in Allaqi Village	4.8	0.54
Men and women at Lake Nasser	9.7	1.20
Men and women in Allaqi Village	10.4	1.26
Age ≥50 years and age <50 years at Lake Nasser	39.8	4.13
Age ≥50 years and age <50 years in Allaqi Village	33.3	3.36
Age <50 years at Lake Nasser and in Allaqi Village	2.0	0.42
Age ≥50 years at Lake Nasser and in Allaqi Village	8.5	1.25

Species recognition among older people (age ≥50 years) was 81.1 % and for younger people (age <50 years) 44.6 %, thus indicating a decrease of 54 %. The reference point of 50 years was chosen because people of 50 years of age or older were expected to have experiences of or memories dating from before the establishment of Lake Nasser and the Nile dams. The difference between older and younger people living at Lake Nasser is even greater (39.8 %) compared to Allaqi Village (33.3 %). In addition to an age effect, we also found a gender effect. The mean difference in the species recognition index between men and women is 10.0 % (9.7 % at Lake Nasser and 10.4 % in Allaqi Village). Finally, we found a location effect demonstrating that the difference in the species recognition index at Lake Nasser is 4.8 % higher compared to Allaqi Village.

**Fig. 2:**
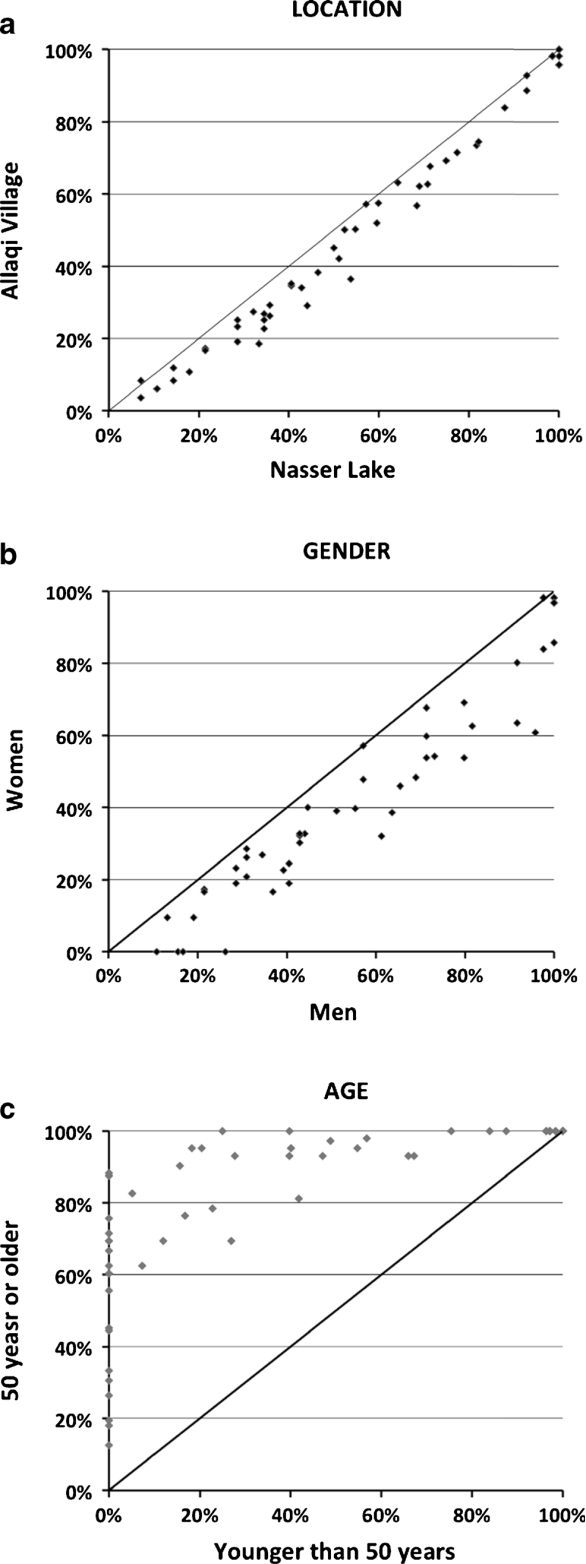
Scatter plots of 61 species (see Appendix) recognized by different groups of respondents. **a** Percentages of men versus women. **b** Percentages of people at Lake Nasser versus Allaqi Village. **c** Percentages of people <50 years versus ≥50 years. The solid line (X=Y) represents equal percentages: no differences between the subgroups. Not all 61 species are visible in these graphs because some points represent multiple species. For example, data point (100 %; 100 %) represents 9 species in each of the graphs, indicating all people in both locations recognize them

The results in Tables 1 and 2 relate to the *R*_*s*_s of the whole set of 61 species. We plotted values of *R*_*s*_ for each separate species vis-à-vis men versus women, for people living at the Lake versus in the Village, and for people younger than 50 versus people 50 years or older (Fig. 2). The solid lines in these graphs represent a situation where there is no difference between the groups (thus Y = X). Clearly, men know more species than women (Table 2), and there is also a difference between people living at Lake Nasser and in Allaqi Village. Both graphs indicate linear and similar trends of *R*_*s*_ between the groups. Thus the difference of knowledge about species between men and women, and between people living at the Lake and in the Village, is more or less similar for all species. This is not the case for the age effect, where we see a strong and nonlinear relationship indicating that a number of species are well known to older people but not at all or only by a few younger people. This is especially the case for species located close to the Y-axis in Fig. 2c.

**Fig. 3:**
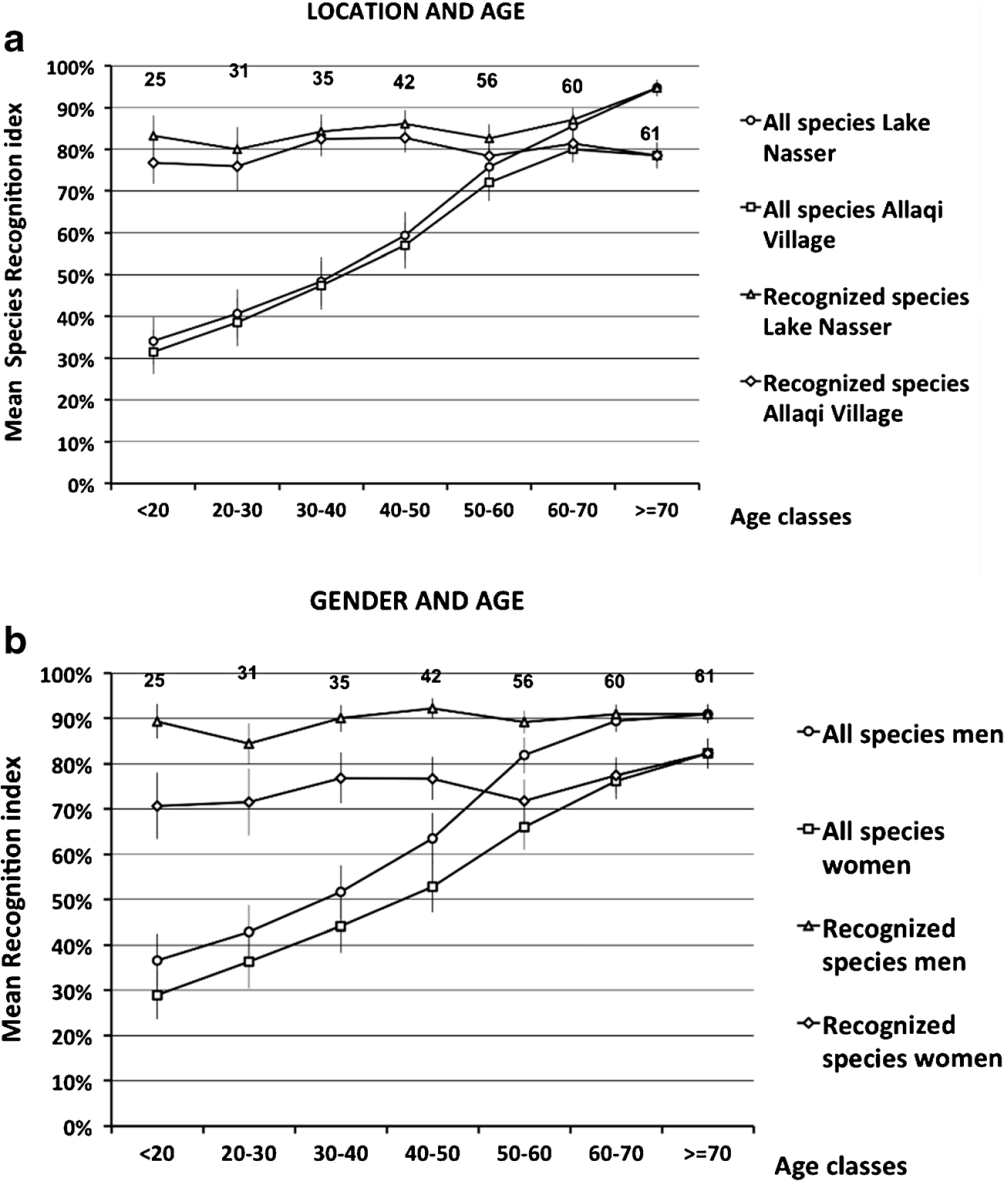
Percentages of species (total 61) recognized in Wadi Allaqi for different age classes. **a** the values of the recognition index, *R*_*s*_, for people at Lake Nasser and in Allaqi Village; **b** for men and women. The two upper graphs at the top in each box are based on Table 3 and refer to the total number of species that are recognized at least by one person in that specific age class; species not recognized by any person in a subset were omitted here and the number in the box represents the total number of species that are recognized. The error bars represent ±1 SEM. Non-parametric testing revealed that all differences are significant except for data point (30–40) in **a**

To better understand the age effect, we derived values of *R*_*s*_ for different age classes with respect to location and gender (Fig. 3a, b). Non-parametric testing shows that all data pairs, except data pair 30–40 years (Fig. 3a, Lake versus Village: *p = 0.061*), differ significantly (*0.000 < p ≤ 0.018*) (Table 2). The value of *R*_*s*_ is in general higher for people living at the Lake than for those living in the Village and higher for men than for women (Fig. 3). In both cases the *R*_*s*_ values increase with age. Because the lines in Fig. 3b are rather parallel we may conclude that loss of knowledge is not gender specific. In these graphs, we also note mean species recognition indexes for subsets of species, in which we omitted species not recognized by anyone in a particular age class. Although these latter indexes still differ between men and women, and between people living at the Lake and in the Village, the age effect disappears completely. The mean values of *R*_*s*_ of these subsets for the different groups (men, women, old, young, people living at the Lake, or in the Village) vary from 71 to 91 % (Table 3). This demonstrates that the reduction of species knowledge over time is the result of the complete disappearance of knowledge of certain species in the younger age groups. It seems that a species is known to a great majority of people in an age class or not at all. Knowledge of species appears to have rather a strong age-group association in the community of Wadi Allaqi Bedouin.

**Table 3:** Species recognition indexes for different age classes based on species recognized within each class at least by one person

Age class	Men	Women	Lake	Village	Number of recognized species in the age classes
<20	89.3 %	70.7 %	83.2 %	76.8 %	25
20–30	84.4 %	71.5 %	80.0 %	75.9 %	31
30–40	90.0 %	76.9 %	84.3 %	82.6 %	35
40–50	92.2 %	76.7 %	86.1 %	82.8 %	42
50–60	89.1 %	71.9 %	82.6 %	78.4 %	56
60–70	91.0 %	77.5 %	87.1 %	81.4 %	60
≥70	91.0 %	82.2 %	94.7 %	78.6 %	61
mean	89.6 %	75.4 %	85.4 %	79.5 %	
SEM	0.9 %	1.6 %	1.8 %	1.0 %	

In conclusion, our data suggest that there is a significant age effect, a gender effect, and a location effect on species recognition, such that older people, men, and people living at the Lake recognize more species as compared to younger people, women, and people living in the Village.

### The Use of Plant Species

**Fig. 4:**
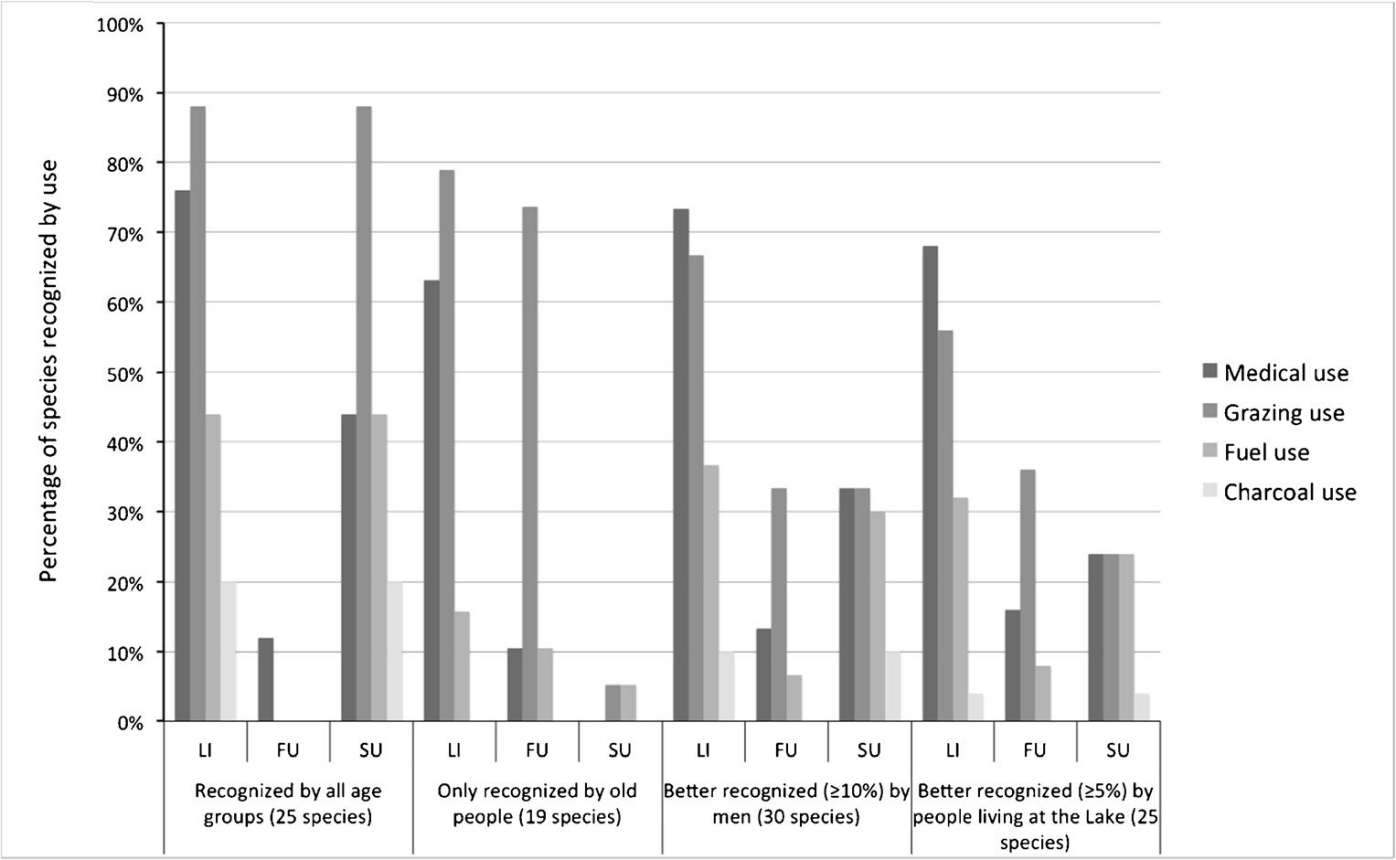
Use recognition of species that were distinguished in the analysis of the recognition factor *R*_*s*_. Legends: LI: reported use in the literature; FU: former use as indicated by the respondents; SU: still in use as indicated by the respondents

Plant species knowledge of indigenous people living in the desert, such as the Bedouin in the area of Wadi Allaqi, is a basic factor in their lives. We therefore expect that for the Bedouin in Wadi Allaqi the dynamics of knowledge of various species would be strongly related to the use of these species. It is especially expected that older people will have more knowledge about species that are no longer in use. We therefore asked them during the interviews about the uses of these species: as medicine, charcoal, fuel, and grazing. We also scanned the literature on their uses (see Tables 4 and 5 in Appendix). With respect to the different uses of plant species, three answers could be distinguished: not used, known to have formerly been used, and still in use (Table 4 in Appendix). We relate these types of answers to four groups of species based on the analysis of the *R*_*s*_ index (Fig. 4; see also Tables 1 and 2): 1) species recognized by people from all age groups, 2) species recognized only by older people (i.e., people 50 years of age and older), 3) species that are better known to men: the difference of *R*_*s*_ > 10 %, and species that are better known to people living at the Lake as compared to people living in the Village: the difference of *R*_*s*_ > 5 %.

Species known to all age groups demonstrate a similar pattern between the species still used and those reported in the literature (Fig. 4). Grazing use was found to be especially important and, to a lesser extent, medicinal use and fuel use. The group of species known only to older people comprises many species formerly used for grazing, many of which are no longer used. These findings indicate that use, and especially grazing use, are related to the preservation of species knowledge among Wadi Allaqi Bedouins.

We should note some limitations of our study. One relates to our limited number of respondents (94), making the subgroups (older people, etc.) rather small. On the other hand, we interviewed about a third of the total population. As indigenous knowledge is often found in rather small populations, we believe that we nevertheless have obtained interesting results with respect to the dynamics of indigenous knowledge under changing socioeconomic circumstances. Another limitation of our study is that, because of the field conditions, we could not prevent people other than the interviewee sometimes participating during interviews, and their input may have influenced on the respondent’s answers. However, we were able to conduct most of the interviews on an individual basis, and we explicitly asked people for their personal opinion; thus, we believe our results are robust in this respect.

## Discussion and Conclusion

The hyper-arid environment of the Wadi Allaqi ecological system has provided a subsistence habitat for its nomadic pastoralists inhabitants for millennia. The Allaqi Bedouin depended to a great extent upon plants for their livelihood, both directly, as part of their diet and fodder for their animals, and indirectly for firewood, shade, construction materials, medicine, etc. Their thorough knowledge of the uses of almost every species of plants growing in the area enabled them to survive in this harsh environment. The main components of their economic system were comprised livestock transhumance, charcoal production and collection of medicinal plants.

This seems to have changed after the construction of the High Dam construction in the 1960s and the creation of Lake Nasser, and later with the establishment of the village in downstream Wadi Allaqi. These developments have drawn many Ababda and Bisharin into semi-permanent residences around the water resource. In addition, since the inundation the presence of both government and private sector institutions and companies in the desert has resulted in numerous employment opportunities for desert residents and there has been an increase in the provision of social services. In this context of new economic opportunities residents are changing their ways of making their living. One of the most significant changes in recent years is the rise of wage labour opportunities for Wadi Allaqi residents. In addition, several agencies have encouraged agricultural activities and have offered material incentives to Allaqi residents. While pastoralism and charcoaling require transhumance, wage labour and agriculture are compatible with a sedentary life.

These dramatic changes are reflected in the decline of traditional knowledge about plants that is especially manifest among younger generations. Older people, men, and people living at the Lake recognized many more species shown them by the interviewers as compared to younger people, women, and people living in the Village. We also found that complete sets of species disappear from recognition in a particular age class but that the recognition index of the remaining species still remains high (around 70–90 %, see Table 3, Fig. 3), indicating a demographic effect. Moreover it appears (see Fig. 4) that species still recognized by all age groups, including younger people, are usually the species that are still used for medicine, grazing, fuel, and charcoal.

Several developments may be related to the loss of species knowledge especially among younger people. First, the establishment of Lake Nasser has led to new and extensive grazing opportunities close to the lake, and to the establishment of Wadi Allaqi village by the lake. Sedentarization is actively encouraged by the Egyptian government (see also, e.g., Baer 1957; Kliot and Medzini 1985), which provides local people with houses, education, and health facilities as attractive alternatives to the nomadic life style. So, it is to be expected that knowledge of desert species declines generally, and particularly among younger people.

Sedentarization may have indirect effects as well. In our research nomadic pastoralism has ceased because of the convenience of grazing opportunities in the vicinity. Livestock grazing is now the responsibility of only one or two persons in a household. Also, other activities such as charcoal production and collection of medicinal plants, have decreased and have a much more marginal role than in former times. Thus differences between age classes in species knowledge and recognition can be explained by the sedentary lifestyle that developed after the construction of Lake Nasser.

We also found that women have less species knowledge than men. This may be related to the traditional Bedouin culture: Because among the Ababda and Bisharin it is often only men who are traveling with taking sheep and goats to distant parts of the desert, and to the markets in Aswan. Women are often not allowed to meet men from outside the family and they cannot travel to the town unaccompanied. They have special tents, the *beit bersh*, where they stay when guests visit. Since women no longer migrate with men to the hills as in the past and because it is generally not possible for them to take advantage of the new jobs in the region, they are forced to stay near or in their houses to take care of their livestock, which is increasingly considered as the women’s responsibility. Thus the sedentarization process may perhaps affect women even more with respect to their species knowledge. However, there is not a significant difference with regard to the role of age in knowledge loss between men and women (Fig. 3b).

In conclusion, we have quantified species knowledge among Bedouin people in Wadi Allaqi and have found a decline of traditional knowledge with respect to plant species, a phenomenon that can be understood by the dramatic environmental and socio-economic changes that has happened in the last 50 years in this area.

## Appendix

**Table 4:** List of 61 species recognized by Bedouin people

Scientific name	Common name	Use according to literature				Use according to respondents			
		M	G	F	C	M	G	F	C
*Aerva javanica* (Burm.f) Juss.ex Schult.	Araa	1	1	0	0	0	2	0	0
*Cymbopogon proximus* (Hochst.) Stapf	Halfa bar	1	1	0	0	2	2	0	0
*Glinus lotoides* L.	Ghobbeira or Toroba	1	1	0	0	1	2	0	0
*Pulicaria crispa* (Forssk.) Oliv.	Kitiye, Kit kaat	1	1	1	0	1	2	2	0
*Senna alexandrina* (Mill.). (syn. *Cassia senna* L.)	Senamekki	1	1	1	0	2	2	2	0
*Sesbania sesban* (L) Merr.	Sesebaan	1	1	1	0	0	2	2	0
*Solenostemma argel* (Del.) Hayne	Argel/Hargel	1	0	0	0	2	0	0	0
*Tamarix nilotica* (Ehrenb.) Bunge	Tarfa or Abal	1	1	1	0	0	2	2	0
*Ziziphus spina-christi* (L.) Desf.	Nabq	1	1	1	0	2	2	2	0
*Acacia raddiana* Savi (Save) Brenan	Sayaal	1	1	1	1	2	2	2	2
*Cyperus pygmaeus* Rottb.	Se’ed/Nigeel	0	1	0	0	0	2	0	0
*Balanites aegyptiaca* (L.) Delile	Hegleeg/Laaloab	1	1	1	1	2	2	2	2
*Citrullus colocynthis* (L.) Schrad	Handal	1	1	0	0	2	2	0	0
*Convolvulus prostratus* Forssk.	Shagaret el-ghazaal	1	0	0	0	2	0	0	0
*Cynodon dactylon* (L.) Pers	Nigeel	1	1	0	0	0	2	0	0
*Najas horrida* A. Br. Ex Magn.	Shilbeika abu Leif	0	1	0	0	0	2	0	0
*Najas marina subsp. armata* (Lindb. f.) Horn	Shilbeika abu Shouk	0	1	0	0	0	2	0	0
*Najas minor* All.	Shilbeika naema	0	1	0	0	0	2	0	0
*Hyoscyamus muticus* (L.)	Sakaran	1	1	0	0	2	2	0	0
*Eragrostis aegyptiaca* (Willd.) Delile	Arbeyaan/Nageela	0	1	0	0	0	2	0	0
*Acacia ehrenbergiana* (Hayne).	Salam	1	1	1	1	2	2	2	2
*Calotropis procera* (Ait.) Ait.	Oshar	1	0	1	0	1	0	2	0
*Crypsis schoenoides* (L.) Lam	Nigeel	0	1	0	0	0	2	0	0
*Acacia tortils* (Forssk.) Hayne	Samra/Samoor	1	1	1	1	0	2	2	2
*Acacia nilotica* L. (Delile)	Qordi, Sant	1	1	1	1	2	2	2	2
*Ricmus communis* L.	Kharwa	1	0	1	0	2	0	2	0
*Pulicaria incisa* (Lam.) DC.	chay gabali	1	0	0	0	2	0	0	0
*Alhagi graecorum* Boiss.	Agool	1	1	1	0	2	2	2	0
*Salvadora persica* L.	Arak	1	1	1	0	2	2	2	0
*Astragalus vogelii* (Webb) Bornm.	Taweel/Qarn	0	1	0	0	0	2	0	0
*Hyphaene thebaica* (L.) Mart.	Doam	1	0	0	0	2	0	0	0
*Fimbristylis bis-umbellata* (Forssk.) Bubani	Hasheesh bahr/Nigeel	0	1	0	0	0	2	0	0
*Medemia argun* (Martius) Wurtt. ex Mart.	Dom el-gabal/Argoon	1	0	0	0	2	0	0	0
*Morettia philaeana* (Del.) DC.	Taaghagha	0	1	0	0	0	2	0	0
*Cleome droserifolia* (Forssk.) Delile	ufiin	1	0	0	0	2	0	0	0
*Psoralea plicata* Delile	Marmid/Marmeed	1	1	0	0	1	2	0	0
*Zilla spinosa* L.	Silla	1	1	1	0	0	2	2	0
*Lotononis platycarpa* (Viv.) Pic Serm.	Oshb	0	1	0	0	0	1	0	0
*Lupinus varius* L. *ssp. orientalis* Frnco et Silva	Tirmis esh-shytaan	1	0	0	0	2	0	0	0
*Leptadenia pyrotechnica* (Forssk.) Decne	Markh	1	1	1	0	0	1	1	0
*Trianthema crystallina* (Forssk.) Vahl	Ararib	0	1	0	0	0	1	0	0
*Capparis decidua* (Forssk.) Edgew.	Tondob	1	0	1	0	1	0	2	0
*Haplophyllum tuberculatum* (Forssk.). Juss	Qarn el-gazal	1	0	0	0	1	0	0	0
*Portulaca oleracea* L.	Rigla	1	1	0	0	1	1	0	0
*Tribulus terrestris* L.	Dreiss	1	1	0	0	0	2	0	0
*Zygophyllum simplex* L.	Garmal	0	1	0	0	0	1	0	0
*Cocculus pendulus* (J.R. and G.Forst.) Diels	Libaakh el-gabal	1	0	1	0	0	0	1	0
*Imperata cylindrica* L.	Halfa	1	1	0	0	0	1	0	0
*Salsola baryosma* (Schult.) Dandy	Khreyt	1	0	1	0	0	0	2	0
*Phragmites australis* (Cav.) Trin ex Steud	Heesh/bousse	1	1	0	0	0	1	0	0
*Panicum turgidum* (Forssk.)	Abu rokba/Thommaam	1	1	0	0	0	1	0	0
*Tribulus mollis* Ehrenb. ex Schweinf.	Qatoob	0	1	0	0	0	1	0	0
*Indigofera hochstetteri* Baker	Tokhayeit	0	1	0	0	0	1	0	0
*Aristida mutabilis* Trin. and Rupr.	Abu-rokrba	0	1	0	0	0	1	0	0
*Astragalus eremophilus* Bioss	Faga’aye	0	1	0	0	0	1	0	0
*Cotula cinerea* Del.	Ribyaan	1	1	0	0	0	1	0	0
*Salsola imbricata* (Forssk.)	Harm	1	1	0	0	0	1	0	0
*Eragrostis ciliaris* (L.) R. Br.	Dabbook	0	1	0	0	0	1	0	0
*Cassia italica* (Mill.) Lam. ex Steud. syn. *Senna italic* Mill.	Sherqaan	1	0	1	0	0	0	1	0
*Crotalaria aegyptiaca* Benth.	Natash	1	1	0	0	0	1	0	0
*Fagonia indica* Burm.f.	Shoka’a	0	1	0	0	0	1	0	0

Legends: M = Medicinal use, G = grazing use, F = fuel use, C = Charcoal use. Numbers in the column Use according to literature: 0 = use not mentioned; 1 = use mentioned. Number in the column “Use according to respondents”: 0 = use not mentioned; 1 = former use; 2 = still in use

**Table 5:** List of 34 species not recognized by respondents

Scientific name	Common name	Use according to literature			
		M	G	F	C
*Abutilon pannosum* (G.Forst. f.) Schltdl.	Loaq	1	0	0	0
*Amaranthus graecizans* L.	Fiss el-kilaab	1	0	0	0
*Aristida adscensionis* L.	Ilaab	0	1	0	0
*Aristida funiculata* Trin. and Rupr.	Qaw	0	1	0	0
*Arnebia hispidissima* (Lehm.) DC	Attan, Attani	1	1	0	0
*Asphodelus fistulosus* L. v. *tenuifolius* Cav.	Busseil/Basal el-khalaa	1	0	0	0
*Caylusea hexagyna* (Forssk.) M.L.Green	Danabaan	0	1	0	0
*Chenopodium murale* L.	Lisaan et-thor	0	1	0	0
*Cistanche phelypaea* (L.) Cout.	Zabb (Zibb) el-ard	1	0	0	0
*Cleome amblycarpa* L.	Shiddiq el-kalb	1	0	0	0
*Cornuulaca monacantha* Del.	Haad	1	0	0	0
*Cucumis prophetarum* Jusl. ap. L.	Heneidlaan	1	0	0	0
*Dichanthium foevulatum* Delile	Seyfoon	0	1	0	0
*Dipterygium glaucum* Decne.	Soffeyrah	0	0	1	0
*Euphorbia granulata* Forssk.	Libbain	1	0	0	0
*Fagonia bruguieri* DC.	Khoshyaat	1	1	0	0
*Fagonia glutinosa* Delile	Medeiheena	0	1	0	0
*Farsetia aegyptia* Turra	Melekiya	0	1	0	0
*Heliotropium bacciferum* Forssk.	Rommaan	1	0	0	0
*Heliotropium supinum* (L.)	Shoak ed-dab’a	1	0	0	0
*Ifloga spicata* (Forssk.) Sch.-Bip.	Kreishit el-gadye	0	1	0	0
*Indigofera argentea* (Burm.)	Damara	0	1	0	0
*Launaea mucronata* (Forssk.) Muschl.	Moraar	1	0	0	0
*Lycium shawii* Roem. Et Sch.	Sahanoon	0	0	1	0
*Maerua crassifolia* (Forssk.)	Margaam	1	1	1	0
*Pergularia tomentosa* L.	Galga	1	0	0	0
*Polycarpaea repens* (Forssk.) Asch. et Schweinf.	Mokor	1	0	0	0
*Polygala erioptera* DC.	Hikal	1	0	0	0
*Rumex vesicarius* L.	Hambeit	1	0	0	0
*Schouwia purpurea* (Forssk.)	Mahad	0	1	0	0
*Stipagrostis plumosa* (L.) Munro ex T. Anders	Nawa beida	0	1	0	0
*Tephrosia apollinea* (Delile) Link	Omay	0	1	0	0
*Tragus berteronianus* Schult	Harroay	0	1	0	0
*Tribulus pentandrus* Forssk.	Shishiq	0	1	0	0

Legends: M = Medicinal use, G = grazing use, F = fuel use, C = Charcoal use. Numbers in the column Use according to literature: 0 = use not mentioned; 1 = use mentioned

## References

[CR1] Ali MM, Dickinson G, Murphy KJ (2000). Predictors of Plant Diversity in a Hyperarid Desert Wadi Ecosystem. Journal of Arid Environments.

[CR2] Ali MA, Badri MA, Moalla SN, Pulford ID (2001). Cycling of Metals in Desert Soils: Effects of *Tamarix nilotica* and Inundation by Lake Water. Environmental Geochemistry and Health.

[CR3] Ayyad MA (1973). Vegetation and Environment of the Western Mediterranean Land of Egypt: The Habitat of Sand-Dunes. Journal of Ecology.

[CR4] Ayyad MA, El-Ghonemy A (1976). Phyto-Sociological and Environmental Gradients in a Sector of the Western Desert of Egypt. Vegetatio.

[CR5] Ayyad, M. A., and Ghabbour, S. I. (1986). Hot deserts of Egypt and Sudan: 149–202. In Evenari, M., and Goodall, D. W. (eds.), Ecosystems of the World, 12B, Hot Desert and Arid Shrublands, B. –Amsterdam.

[CR6] Baer G (1957). Some Aspect of Bedouin Sedentarization in 19th Century Egypt. Die Welt des Islams, New Series.

[CR7] Badri M, Hamed A (2000). Nutrient Value of Some Plants in an Extremely Arid Environment (Wadi Allaqi Biosphere Reserve, Egypt). Journal of Arid Environments.

[CR8] Badri MA, Pulford ID, Springuel I (1996). Supply and Accumulation of Metals in Two Egyptian Desert Plant Species Growing on Wadi-Fill Deposits. Journal of Arid Environments.

[CR9] Belal, A. E., Leith, B., Solway, J., and Springuel, I. (1998). Environmental Valuation and Management of Plants in Wadi Allaqi, Egypt. Final Report Submitted to International Development Research Centre (IDRC) Canada.

[CR10] Belal, A., Briggs, J., Sharp, J., and Springuel, I. (2009). Bedouins by the Lake: Environment, Change, and Sustainability in Southern Egypt. AmerUniv. in Cairo Pr. 184 pp.

[CR11] Boulos L (1999). Flora of Egypt, 1 (Azollaceae–Oxalidaceae).

[CR12] Boulos L (2000). Flora of Egypt, 2 (Geraniaceae–Boraginaceae).

[CR13] Boulos L (2002). Flora of Egypt, 3 (Verbenaceae–Compositae).

[CR14] Briggs J, Dickinson G, Murphy K, Pulford I, Belal AE, Moalla S, Springuel I, Ghabbour SI, Mekki AM (1993). Sustainable Development and Resource Management in Marginal Environments: Natural Resources and Their use in the Wadi Allaqi Region of Egypt. Applied Geography.

[CR15] Briggs J, Badri M, Mekki AM (1999). Indigenous Knowledges and Vegetation use Among Bedouin in the Eastern Desert of Egypt. Applied Geography.

[CR16] Briggs J, Sharp J, Hamed N, Yacoub H (2003). Changing women’s Roles, Changing Environmental Knowledges: Evidence from Upper Egypt. Geographical Journal.

[CR17] Briggs J, Sharp J, Yacoub H, Hamed N, Roe A (2007). The Nature of Indigenous Knowledge Production: Evidence from Bedouin Communities in Southern Egypt. Journal of International Development.

[CR18] Dubroeucq D, Livenais P (2004). Land Cover and Land use Changes in Relation to Social Evolution—a Case Study from Northern Chile. Journal of Arid Environments.

[CR19] EEAA. (2005). Unpublished report. EEAA, Egyptian Environmental Affairs Agency, Ministry of State for Environmental Affairs. NCS, Nature Conservation Sector. WABR, Wadi Allaqi Biosphere Reserve. Aswan, Egypt.

[CR20] EEAA. (2012). EEAA, Egyptian Environmental Affairs Agency, Ministry of State for Environmental Affairs. NCS, Nature Conservation Sector. WABR, Wadi Allaqi Biosphere Reserve. Aswan, Egypt.

[CR21] Fossati J, Pautou G, Peltier J (1998). Wadi Vegetation of North Eastern Desert of Egypt. Feddes Repertorium.

[CR22] Goodman SM, Hobbs JJ (1988). The Ethnobotany of the Egyptian Eastern Desert: A Comparison of Common Plant Usage Between Two Culturally Distinct Bedouin Groups. Journal of Ethnopharmacology.

[CR23] Hamed A, El-Emary N (1999). Triterpene Saponins from *Glinus lotoides* var. *dictamnoides*. Phytochemistry.

[CR24] Hamed A, Springuel I, El-Emary N, Mitome H, Miyaoka H, Yamada Y (1996). Triterpenoidal Saponin Glycosides from *Glinus lotoides* var. *dictamnoides*. Phytochemistry.

[CR25] Hamed AI, El-Emary NA, Springuel I, Mitome H, Myoika H, Yamada Y (1997). A Phenolic Cinnamate Dimer from *Psoralea plicata* Del. Phytochemisty.

[CR26] Hamed A, Springuel I, El-Emary N (1999). Benzofuran Glycosides from *Psoralea plicata* Seeds. Phytochemistry.

[CR27] Hamed A, Oleszek W, Stochmal A, Pizza C, Piacente S (2004). Steroidal Saponins from the Aerial Parts of *Tribulus pentandrus* Forssk. Phytochemistry.

[CR28] Hassan HA, Hamed A, El-Emary N, Springuel I, Mitome H, Miyaoka H (2001). Pregnane Derivatives from *Solenostemma argel*. Phytochemistry.

[CR29] Hobbs JJ (1989). Bedouin Life in the Egyptian Wilderness.

[CR30] Ibrahim H, Baker WJ (2009). *Medemia argun*. PALMS.

[CR31] Kassas M (1952). Habitats and Plant Communities in the Egyptian Deserts. I. Introduction. Journal of Ecology.

[CR32] Kassas M, Girgis WA (1970). Habitat and Plant Communities in the Egyptian Desert: VII. Geographical Facies of Plant Communities. Journal of Ecology.

[CR33] Käyhkö N, Fagerholm N, Asseid B, Mzee A (2011). Dynamic Land use and Land Cover Changes and Their Effect on Forest Resources in a Coastal Village of Matemwe, Zanzibar, Tanzania. Land Use Policy.

[CR34] Klintenberg P, Seely M, Christiansson C (2007). Local and National Perceptions of Environmental Change in Central Northern Namibia: Do They Correspond?. Journal of Arid Environments.

[CR35] Kliot N, Medzini A (1985). Bedouin Settlement Policy in Israel, 1964–1982: Another Perspective. Geoforum.

[CR36] Mekki, A. M., and Briggs, J. (1991). The Economic System of Wadi Allaqi, Allaqi Project Working Paper No 11. University of Glasgow and Faculty of Science in Aswan, Assiut University.

[CR37] Mohamed, A. S. I., Mekki, A. M., and Briggs, J. (1991). The Social and Demographic Structure of Wadi Allaqi. Allaqi Project working Papers, No. 16. University of Glasgow and Faculty of Science in Aswan, Assiut University.

[CR38] Noy-Meir I (1973). Desert Ecosystems: Environment and Producers. Annual Review of Ecology and Systematics.

[CR39] Pulford I, Murphy K, Dickinson G, Briggs J, Springuel I (1992). Ecological Resources for Conservation and Development in Wadi Allaqi Egypt. Botanical Journal of the Linnean Society.

[CR40] Radwan U (2007). Photosynthetic and Leaf Anatomical Characteristics of the Drought-Resistant *Balanites aegyptiaca* (L.) Del. Seedlings. American-Eurasian Journal of Agricultural and Environmental Sciences.

[CR41] Rohde RF, Hoffman MT (2012). The Historical Ecology of Namibian Rangelands: Vegetation Change Since 1876 in Response to Local and Global Drivers. Science of the Total Environment.

[CR42] Shaltout KH, Sheded MG, Salem AI (2009). Population Structure of Common Shrubs and Trees in Wadi Allaqi Biosphere Reserve, South-East Egypt. Feddes Repertorium.

[CR43] Shaltout KH, Sheded MG, Salem AI (2010). Vegetation Spatial Heterogeneity in a Hyper Arid Biosphere Reserve Area in North Africa. Acta Botanica Croatia.

[CR44] Sharp J, Briggs J, Yacoub H, Hamed N (2003). Doing Gender and Development: Understanding Empowerment and Local Gender Relations. Transactions. Institute of British Geographers.

[CR45] Sheded, M. G. (1992). Environment and Vegetation in the South Eastern Desert, Egypt. PhD Thesis, Faculty of Science at Aswan, Assiut University.

[CR46] Sheded MG (1998). Vegetation Pattern Along an Edaphic and Climatic Gradient in the South-Eastern Desert of Egypt. Feddes Repert.

[CR47] Sheded MG, Pulford ID, Hamed AI (2006). Presence of Major and Trace Elements in Seven Medicinal Plants Growing in the South-Eastern Desert, Egypt. Journal of Arid Environments.

[CR48] Solway, J., and Mekki, A. M. (1999). Socio-economic system of Wadi Allaqi. Project Working Paper No 33. Aswan, University of South Valley.

[CR49] Springuel, I. (1994). Plant Ecology of Wadi Allaqi and Lake Nasser No. 4: Basis for economic utilization and conservation of vegetation in Wadi Allaqi conservation area, Egypt. Published by UESD, South Valley University, Aswan, Egypt.

[CR50] Springuel, I., and Belal, A. (2001). A Case Study On Ecotourism In The Wadi Allaqi Biosphere Reserve. Report. UNESCO-Cousteau Ecotechnie Chair on Environment and Sustainable Development at the Unit of Environmental Studies and Development (UESD), South Valley University. Submitted to UNESCO, Division of Ecological Science, Egypt.

[CR51] Springuel I, Mekki AM (1994). Economic Value of Desert Plants: Acacia Trees in Wadi Allaqi Biosphere Reserve. Environmental Conservation.

[CR52] Springuel I, Murphy KJ (1990). Euhydrophytes of Egyptian Nubia. Aquatic Botany.

[CR53] Tackholm, V. (1974). Student’s Flora of Egypt. Cairo University Publication.

[CR54] White GF (1988). The Environmental Effects of the High Dam at Aswan. Environment.

[CR55] Yacoub H (2009). *Najas spp*. Growth in Relation to Environmental Factors in Wadi Allaqi (Nasser Lake, Egypt). Transylvanian Review of Systematical Ecological Research.

[CR56] Yacoub, H. (2012) Agropastoralism as Strategy for Sustainable Conservation and Livelihood in Wadi Allaqi Biosphere Reserve, South Eastern Desert, Egypt. The Rufford Small Grants Foundation, London.

